# Development and characterization of a monoclonal antibody against Pseudorabies virus glycoprotein B and its application in tracking viral infection

**DOI:** 10.3389/fmicb.2026.1875884

**Published:** 2026-06-17

**Authors:** Hai-Ming Wang, Yue Sun, Yu Wang, Ping-Yu Huang, Peng Liu, Yanhe Zhang, Yan-Dong Tang

**Affiliations:** 1 Jiangsu Agri-Animal Husbandry Vocational College, Jiangsu, China; 2 State Key Laboratory of Animal Disease Control and Prevention, Harbin Veterinary Research Institute of Chinese Academy of Agricultural Sciences, Harbin, China

**Keywords:** glycoprotein B (gB), immunofluorescence assay, monoclonal antibody, Pseudorabies virus (PRV), viral infection tracking

## Abstract

Pseudorabies virus (PRV) is a swine herpesvirus that causes severe economic losses in the global pig industry. Glycoprotein B (gB) is a highly conserved envelope glycoprotein of PRV and plays an essential role in viral entry, cell fusion, and antibody induction. Here, the extracellular domain of gB from PRV HeN1 strain was expressed in CHO cells and purified. After mouse immunization and hybridoma fusion, a gB-specific monoclonal antibody 1G4 was identified via ELISA and IFA. 1G4 presented high specificity, good reactivity and broad cross-recognition against multiple PRV strains. FITC-labeled 1G4, combined with a gD-targeted mAb, was applied to visualize PRV adsorption and internalization in HeLa cells by confocal microscopy at fixed time points. In conclusion, this novel gB mAb serves as a reliable reagent for PRV mechanism research and immunological detection.

## 1 Introduction

Pseudorabies virus (PRV), a member of the Alphaherpesvirinae subfamily, is the etiological agent of Aujeszky’s disease, characterized by high mortality in newborn piglets, reproductive failure in sows, and respiratory distress in growing pigs (Davison et al., 2009; Wang et al., 2026). Pigs serve as the primary natural host for PRV, which also infects a broad range of other mammals as dead-end hosts (Sawitzky, 1997; Mettenleiter, 2000). Since the emergence of PRV variants in China since 2011, traditional Bartha K61-based vaccines have shown reduced protective efficacy, leading to persistent outbreaks and increased risk of cross-species transmission (An et al., 2013; Luo et al., 2014; Yu et al., 2014; Tong et al., 2015). PRV has been reported to infect humans occasionally, raising public health concerns (Ai et al., 2018; He et al., 2019; Wong et al., 2019). Although live-attenuated vaccines (Tang et al., 2016, 2018), and mRNA vaccines have been developed (Sun et al., 2025), and neutralizing antibodies have shown therapeutic potential (Sun et al., 2026), there is still an urgent need for reliable reagents for viral detection, antigenic analysis, and mechanistic studies (Na et al., 2025a). Such tools are of great significance for the prevention and control of PRV. As the most conserved envelope glycoprotein in herpesviruses, gB is a class III fusion protein essential for viral penetration and cell-to-cell spread (Wang et al., 2025). gB mediates membrane fusion between the viral envelope and host cell membrane, as well as fusion between infected and adjacent uninfected cells, forming syncytia (Chen et al., 2022; Huang et al., 2024; Rao et al., 2024). Moreover, gB is a dominant antigen that induces robust humoral and cellular immune responses, making it a key target for diagnostic reagents, neutralizing antibody development, and vaccine design (Sun et al., 2016; Tan et al., 2021; Bo and Li, 2022; Zheng et al., 2022).

Monoclonal antibodies (mAbs) offer high specificity, homogeneity, and sustainable production, which are irreplaceable in virology research. Specific mAbs against viral glycoproteins can be used for antigen detection, protein localization, epitope mapping, neutralization assay, and dynamic tracking of viral infection. Although several antibodies against PRV proteins have been reported, a well-characterized mAb targeting gB that enables live-cell tracking of viral entry remains in demand.

In this study, we established a mammalian expression system to produce recombinant PRV gB, generated a highly specific mAb 1G4, and comprehensively evaluated its reactivity, specificity, and cross-reactivity. Furthermore, we developed a FITC-labeled 1G4 probe and applied it to visualize PRV adsorption and internalization. This study provides a validated antibody tool for PRV basic research and diagnostic applications.

## 2 Materials and methods

### 2.1 Cells, viruses, plasmids, and antibodies

SP2/0 myeloma cells, HEK293T cells, Vero E6 cells, and HeLa cells were all preserved in our laboratory. Pseudorabies virus (PRV) strains HeN1 (GenBank Accession No. KP098534), SC (GenBank Accession No. KT809429), TJ (GenBank Accession No. KJ789182), and Bartha K61 (GenBank Accession No. JF797217) were preserved in our laboratory (Sun et al., 2025). Plasmids pCAGGS-HA-gB and pb513B were also preserved in our laboratory (Yang et al., 2021; Wang et al., 2022, 2023; Huang et al., 2024; Rao et al., 2024). The PRV gD-specific monoclonal antibody (mAb) 1D11 was described in our previous study (Na et al., 2025a).

### 2.2 Construction of a CHO cell line stably expressing gB protein

The extracellular domain of the gB gene from PRV HeN1 strain was codon-optimized, and a Kozak sequence and a tPA signal peptide sequence were added to its N-terminus before cloning into the pb513B vector, which contains a 6 × His tag. A stable CHO cell line expressing the gB protein was then established as previously described (Sun et al., 2025). Briefly, pb513B-gB was co-transfected with a PB transposase helper plasmid (System Biosciences, USA) into CHO cells, followed by selection with puromycin (Gibico, USA) to obtain a stable CHO cell line expressing the recombinant gB protein.

### 2.3 Purification and verification of recombinant gB protein

Cell supernatants containing the gB protein were collected and purified using Ni-NTA affinity chromatography resin (GenScript, USA) according to the manufacturer’s instructions. Western blotting analysis of the recombinant gB protein was performed as previously described (Sun et al., 2025). Briefly, the gB protein was separated by SDS-PAGE and transferred to a PVDF membrane, which was blocked with 5% non-fat milk for 1 h. Subsequently, the membrane was incubated overnight at 4 °C with an anti-His monoclonal antibody (Proteintech, USA) or mouse positive serum. After three washes with PBST, the membrane was incubated with DyLight 800-labeled goat anti-mouse IgG antibody (KPL, USA) for 1 h at room temperature (Zhang et al., 2021, 2022). Finally, protein bands were detected using an Odyssey CLX imaging system. The protein concentration was determined using a BCA Protein Assay Kit (Solarbio, China), and the protein was stored at −80 °C until use.

### 2.4 Mouse immunization and specific antibody detection

Mice were immunized with the purified gB protein (100 μg per mouse) three times at 14-days intervals, with mice immunized with PBS serving as negative controls. For the primary immunization, the protein was emulsified with Freund’s complete adjuvant (Sigma, USA). For the subsequent booster immunizations, Freund’s incomplete adjuvant (Sigma, USA) was used for emulsification. One week after the third immunization, blood was collected, and serum was separated. Antibody titers were detected by indirect enzyme-linked immunosorbent assay (ELISA). Briefly, 96-well ELISA plates were coated with the purified gB protein (100 ng per well) overnight at 4 °C. After washing with PBST, the plates were blocked with 3% BSA for 2 h. Serially diluted mouse serum (1:400 to 1:102,400; 100 μL per well) was added and incubated at 37 °C for 1 h. After washing with PBST, HRP-conjugated goat anti-mouse antibody (1:10,000, Zhongshan Golden Bridge, China) was added and incubated at 37 °C for 45 min. Following another wash with PBST, TMB chromogenic solution (100 μL per well) was added, and the plates were incubated at room temperature in the dark for 10 min. The reaction was terminated with 0.5 M hydrochloric acid, and the absorbance at 450 nm was measured within 10 min. The S/N ratio was calculated as the OD_450__*nm*_ value of the sample well (S) divided by the OD_450__*nm*_ of the negative control well (N). Samples with S/N > 2.1 were considered positive. Five mice immunized with gB protein were used as five biological replicates, and each sample was assayed in two technical replicates. The antibody titer determined by endpoint dilution.

### 2.5 Preparation of monoclonal antibodies

Mice with qualified serum titers were selected for booster immunization, and splenocytes were collected 3 days later for fusion with myeloma cells. Briefly, splenocytes were collected under sterile conditions and fused with SP2/0 myeloma cells using PEG1450 (Sigma-Aldrich, USA). The fused cells were cultured in DMEM medium supplemented with 20% FBS and 1% HAT (Sigma-Aldrich, USA). After 7–10 days, positive clones reactive with the gB protein were screened by indirect ELISA. To ensure the monoclonality and stability, the obtained positive clones were subcloned three times by the limiting dilution method. In addition, the reactivity of the monoclonal antibody supernatants with the eukaryotic expression plasmid (pCAGGS-HA-gB) and different PRV strains (HeN1, TJ, SC, and Bartha K61) was evaluated by indirect immunofluorescence assay (IFA).

### 2.6 Plasmid transfection and virus infection

HEK293T cells were seeded into 12-well plates. When the cell confluency reached 70%–80%, pCAGGS-HA-gB was transfected into the cells using PEI transfection reagent. After 6–8 h of transfection, the medium was replaced with fresh medium, and IFA was performed after 24 h of culture.

Vero E6 cells were seeded into 12-well plates. When the cell confluency reached 90%–100%, the cells were infected with PRV strains (HeN1, TJ, SC, or Bartha K61) at a multiplicity of infection (MOI) of 0.01. When obvious cytopathic effect (CPE) was observed, the cells were fixed with 4% paraformaldehyde (PFA) for IFA.

### 2.7 Indirect immunofluorescence assay (IFA)

At 24 h after transfection of HEK293T cells or when obvious CPE appeared in PRV-infected Vero E6 cells, the cells were fixed with 4% paraformaldehyde at room temperature for 30 min, followed by permeabilization with 0.25% Triton X-100 (Beyotime, China) at 4 °C for 10 min. After three washes with PBS, the cells were blocked with 2% BSA at 37 °C for 30 min. After washing with PBS, the cells were incubated with monoclonal antibody supernatant, SP2/0 supernatant, or mouse anti-HA antibody (Proteintech, USA) at 37 °C for 1 h. Following three washes with PBS, the cells were incubated with goat anti-mouse fluorescent secondary antibody (1:200, Sigma, USA) at 37 °C for 1 h. Cell nuclei were stained with 4′,6-diamidino-2-phenylindole (DAPI). Finally, fluorescent signals were detected by microscopy.

### 2.8 Preparation and purification of ascites

Ten-weeks-old SPF-grade BALB/c mice were pre-sensitized by intraperitoneal injection of sterile paraffin oil to create an *in vivo* environment conducive to hybridoma cell proliferation and ascites production. One week after sensitization, each mouse was intraperitoneally inoculated with the hybridoma cell line (1.0∼3.0 × 10^6^ cells per mouse). After 7 days of routine feeding, mouse ascites was collected by abdominal paracentesis. The collected ascites was centrifuged at 10,000 × *g* for 10 min at low temperature to fully remove cell debris, fat globules, and insoluble impurities, and the clear supernatant fraction was collected. Specific monoclonal antibodies in the supernatant were purified by affinity chromatography using Protein A/G magnetic beads (GenScript, USA) according to the manufacturer’s instructions.

### 2.9 FITC labeling of mAbs

Fluorescein labeling of antibodies was performed using an FITC conjugation kit (Sangon Biotech, China) strictly according to the manufacturer’s instructions. During the labeling process, purified antibody (500 μl, 2 mg/ml) and FITC solution (10 μL, 5 mg/ml) were added to a 1.5 ml centrifuge tube. The mixture was incubated at 37 °C in the dark for 90 min on a rotary shaker to ensure efficient conjugation of fluorescein to the antibody. After incubation, unbound free FITC and small-molecule impurities in the system were removed using a desalting column to obtain the purified FITC-labeled antibody. The labeled antibody solution was stored at 4 °C in the dark.

### 2.10 Application of mAb-FITC in PRV adsorption and entry

To evaluate the application of FITC-labeled antibodies in the PRV adsorption stage, PRV HeN1 strain (MOI = 10) was added to pre-cooled HeLa cells and incubated at 4 °C for 2 h to allow virus adsorption. After washing with PBS, the cell membranes were stained with WGA (1:1000, Thermo Fisher, USA) at 4 °C for 10 min. After washing, the cells were fixed and blocked, followed by incubation with mAb 1D11 (a PRV gD-specific antibody) at 37 °C for 1 h. After washing with PBS, Alexa Fluor 568-labeled anti-mouse IgG (Thermo Fisher, USA) was added and incubated at 37 °C for 1 h. Following another wash with PBS, FITC-labeled gB monoclonal antibody (mAb 1G4-FITC, 1:500) was added and incubated at 37 °C for 1 h. Subsequently, cell nuclei were stained with DAPI, and virus particles bound to HeLa cells were observed by confocal microscopy.

To evaluate the application of FITC-labeled antibodies in the PRV entry stage, PRV HeN1 strain (MOI = 10) was added to pre-cooled HeLa cells and adsorbed at 4 °C for 2 h. After washing the cells, fresh pre-warmed medium was added, and the cells were transferred to 37 °C for 2 h to allow internalization. After washing with PBS, the cell membranes were stained with WGA at 37 °C for 5 min. After washing, the cells were fixed, permeabilized, and blocked, then incubated with mAb 1D11 at 37 °C for 1 h. After washing with PBS, Alexa Fluor 568-labeled anti-mouse IgG was added and incubated at 37 °C for 1 h. Subsequently, mAb 1G4-FITC (1:500) was added and incubated at 37 °C for 1 h. Finally, internalized viruses in HeLa cells were observed by confocal microscopy.

### 2.11 Ethical statement

Experiments using SPF-grade female BALB/c mice (6–8 weeks old) were conducted at Jiangsu Agri-animal Husbandry Vocational College. The experimental protocol was reviewed and approved by the Ethics Committee of Jiangsu Agri-animal Husbandry Vocational College (Approval No.: jsahvc-2026-49).

## 3 Results

### 3.1 Expression and purification of recombinant PRV gB protein

To obtain specific antibodies against PRV gB protein, we first expressed and purified the recombinant PRV gB protein. The extracellular domain of PRV HeN1 strain gB was codon-optimized according to CHO cell codon preference, then cloned into the pb513B vector with a C-terminal 6 × His tag for easy purification and identification.

The recombinant pb513B-gB plasmid was transfected into CHO cells via liposome-mediated transfection. After puromycin selection, stable cell lines stably expressing recombinant gB were obtained as previously described (Sun et al., 2025). The recombinant gB protein was purified from serum-free cell culture supernatants by Ni-NTA affinity chromatography, relying on specific binding between the 6 × His tag and nickel ions on the resin.

**FIGURE 1:**
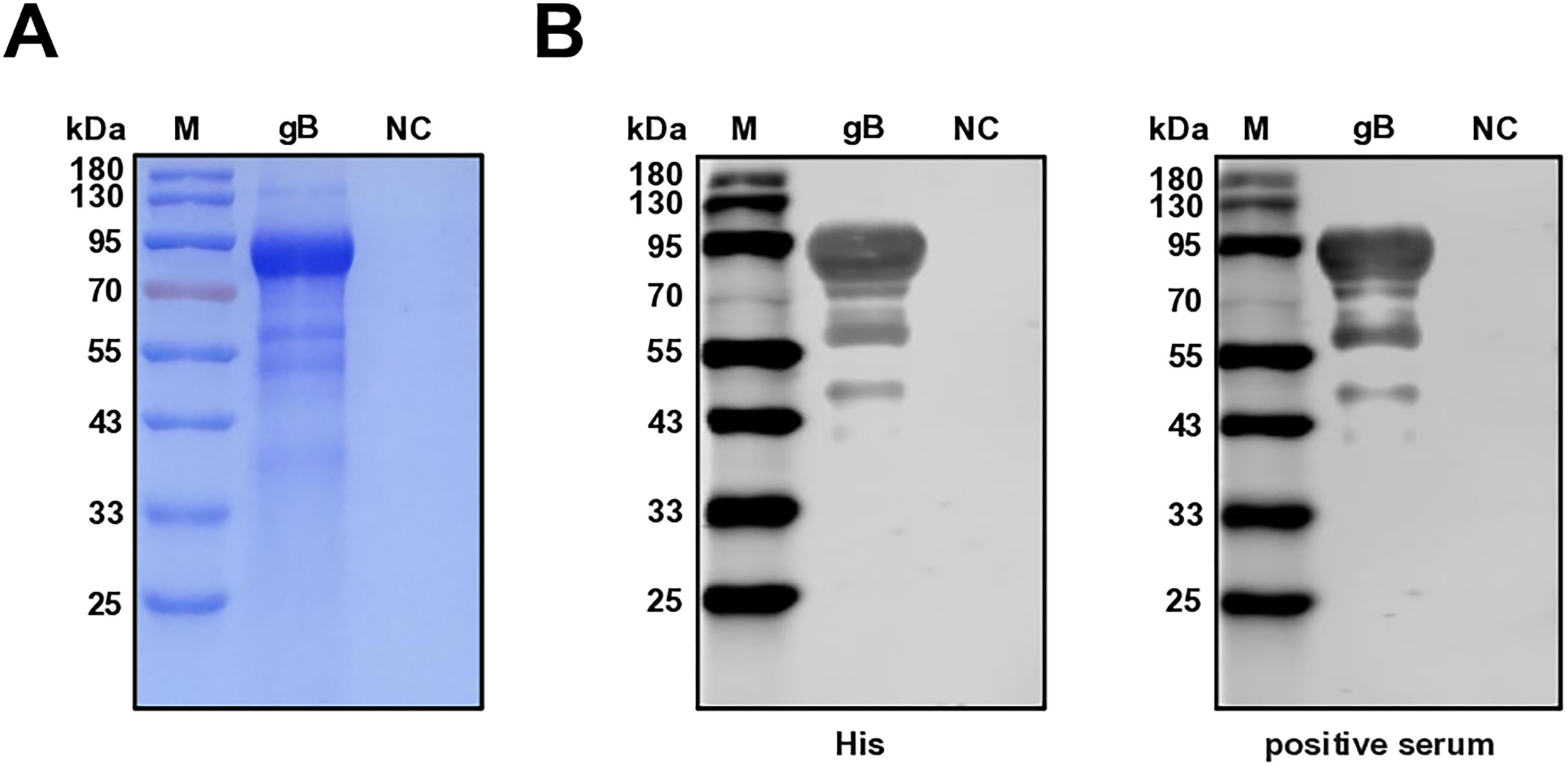
Purification and identification of recombinant PRV gB protein. **(A)** SDS-PAGE analysis of purified gB protein. M, protein molecular weight marker; gB, purified recombinant gB from stable CHO cell supernatant; NC, supernatant from non-transfected CHO cells. **(B)** Western blotting identification of gB protein. Left panel: probed with anti-His tag monoclonal antibody; right panel: probed with gB-immunized mouse positive serum. M, protein marker; gB, target protein; NC, negative control.

SDS-PAGE analysis showed a clear target band at the expected molecular weight (∼80 kDa) with high purity, while no corresponding band was observed in non-transfected CHO cell supernatant (negative control, NC) (Figure 1A). Western blotting with anti-His antibody and PRV gB-positive mouse serum both specifically recognized the purified protein, confirming correct expression and good antigenicity, which laid a foundation for subsequent monoclonal antibody preparation (Figure 1B).

### 3.2 Preparation of PRV gB-specific monoclonal antibodies

**FIGURE 2:**
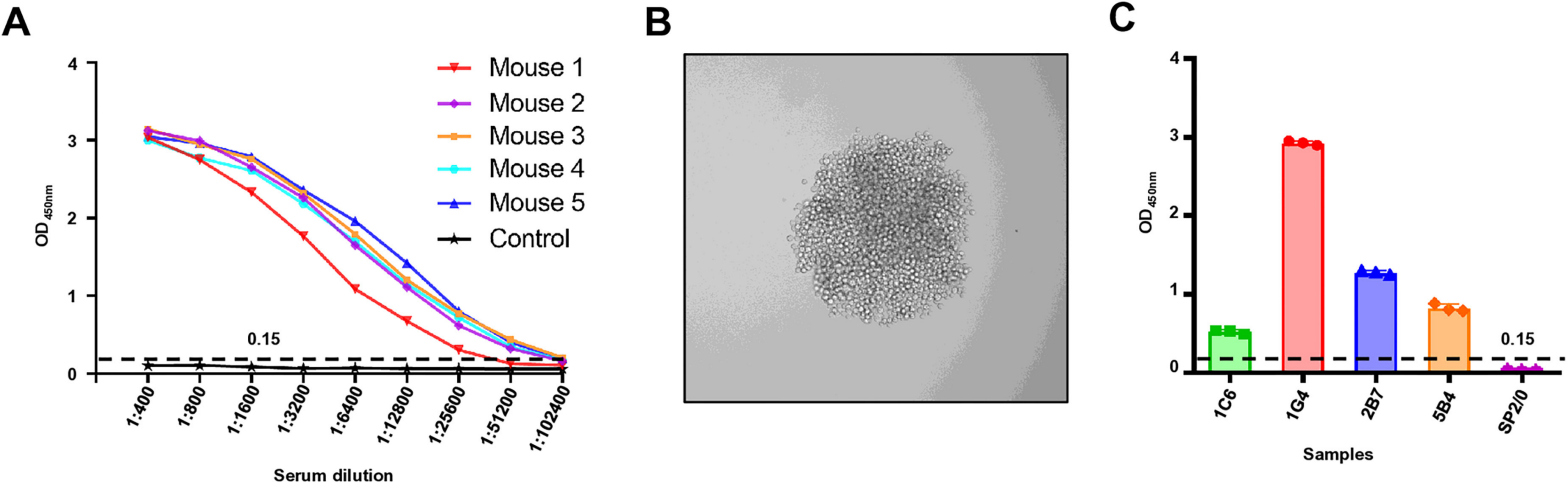
Preparation and screening of gB-specific monoclonal antibodies. **(A)** Serum antibody titers of gB-immunized mice detected by indirect ELISA. Mouse 1–5: mice immunized with recombinant gB; Control: mice injected with PBS. Five biological replicates (gB-immunized mice), two technical replicates per sample. **(B)** Morphology of hybridoma cell clones after three rounds of limiting dilution subcloning. **(C)** Reactivity of hybridoma clones (1C6, 1G4, 2B7, 5B4) and SP2/0 negative control detected by indirect ELISA, three technical replicates per sample.

Using hybridoma technology, 6–8-weeks-old female BALB/c mice were immunized three times with purified recombinant gB protein at 2-weeks intervals. The first immunization used gB mixed with complete Freund’s adjuvant to activate the immune system, and the latter two used incomplete Freund’s adjuvant to enhance antibody titer. Indirect ELISA 1 week after the third immunization showed all immunized mice produced high-titer anti-gB antibodies (>1:51200), while control mice had no specific response (Figure 2A). The mouse with the highest titer was selected as the spleen cell donor to ensure high-affinity B lymphocytes.

Splenocytes from the selected mouse were fused with SP2/0 myeloma cells using PEG (Na et al., 2025a). After HAT selection and three rounds of limiting dilution subcloning, four stable hybridoma clones (1C6, 1G4, 2B7, 5B4) were obtained. Single clones showed good morphology and viability (Figure 2B). After 10 continuous passages, indirect ELISA confirmed 1G4 maintained stable high reactivity, so it was selected for further characterization (Figure 2C).

### 3.3 Reactivity identification of mAb 1G4

**FIGURE 3:**
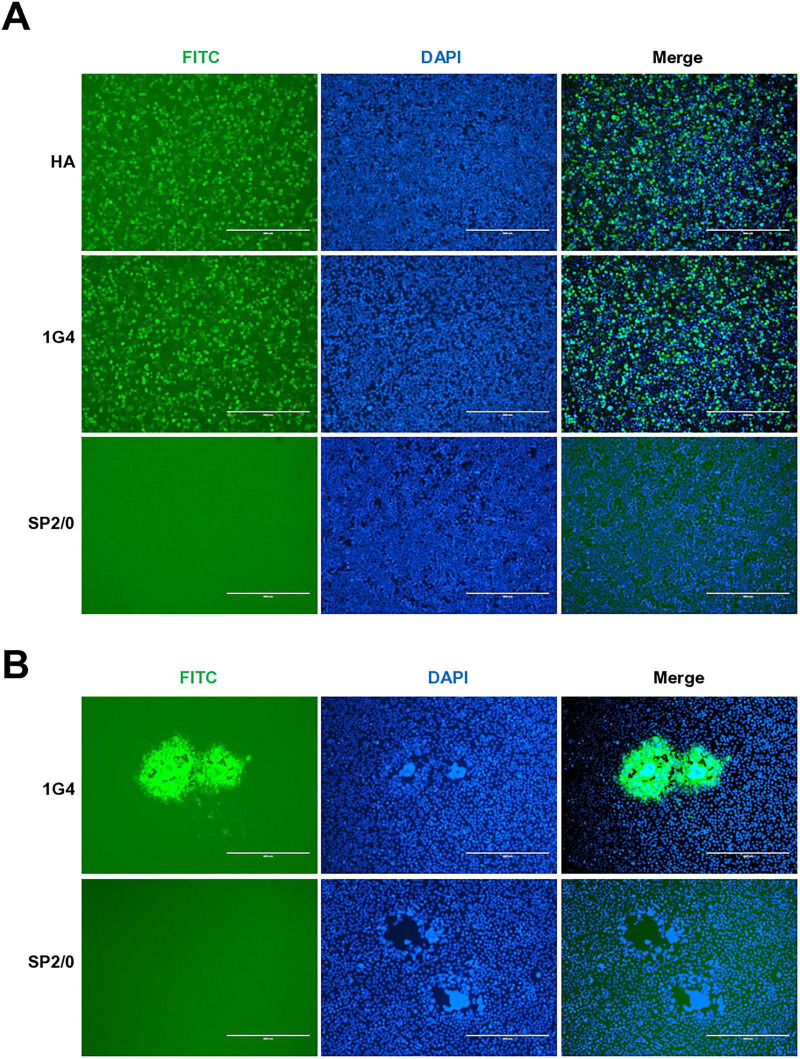
Specific reactivity of mAb 1G4 analyzed by indirect immunofluorescence assay. **(A)** IFA results of HEK293T cells transfected with pCAGGS-HA-gB. 1G4: mAb 1G4 cell supernatant; SP2/0: negative control supernatant; HA: anti-HA antibody positive control; FITC: green fluorescence; DAPI: nuclear staining; Merge: merged fluorescence image. Scale bar = 400 μm. **(B)** IFA results of Vero E6 cells infected with PRV HeN1 strain. 1G4: mAb 1G4 supernatant; SP2/0: negative control. Scale bar = 400 μm.

Indirect immunofluorescence assay (IFA) was performed on two cell models to verify mAb 1G4 specificity: HEK293T cells transfected with gB plasmid (for recombinant gB recognition) and PRV-infected Vero E6 cells (for native gB recognition). HEK293T cells transfected with pCAGGS-HA-gB showed strong green fluorescence when incubated with mAb 1G4 supernatant, consistent with the anti-HA positive control, while no fluorescence was detected in SP2/0 supernatant control (Figure 3A), indicating mAb 1G4 specifically recognizes recombinant gB. Next, Vero E6 cells infected with PRV HeN1 showed specific fluorescence with mAb 1G4, while uninfected cells had no fluorescence (Figure 3B). These results confirm mAb 1G4 specifically recognizes both recombinant and native gB proteins, supporting its application in PRV research and detection.

### 3.4 Cross-reactivity of mAb 1G4 with different PRV strains

**FIGURE 4:**
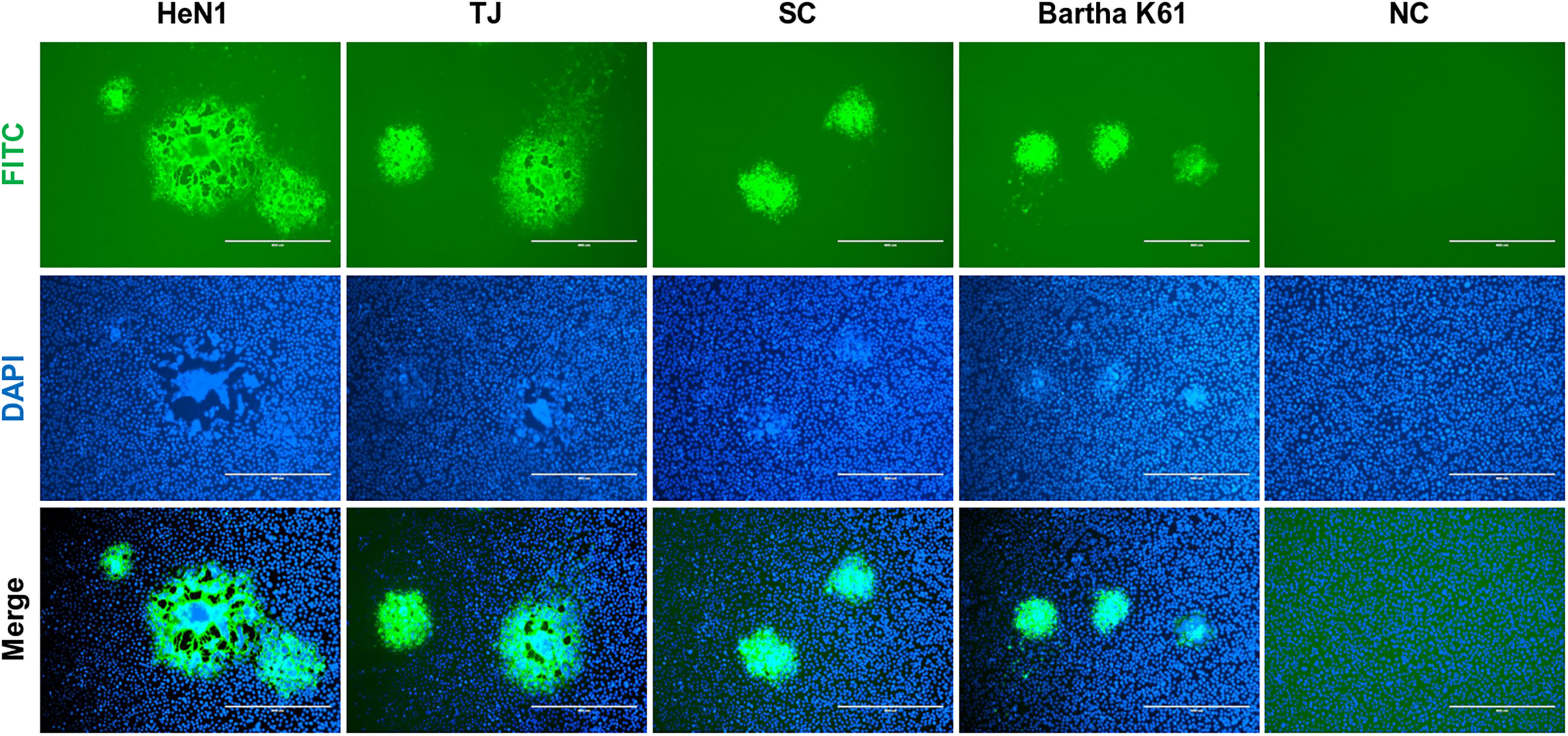
Cross-reactivity of mAb 1G4 with different PRV strains. IFA images of Vero E6 cells infected with HeN1, TJ, SC, and Bartha K61 strains, respectively. FITC: specific fluorescence of mAb 1G4; DAPI: blue nuclear staining; Merge: merged image; NC: uninfected cell control. Scale bar = 400 μm.

To evaluate mAb 1G4 reactivity breadth, IFA was performed on Vero E6 cells infected with four representative PRV strains covering different genetic branches: Bartha K61 (genotype I, attenuated vaccine strain) and HeN1, TJ, SC (genotype II; SC is a classic strain, HeN1 and TJ are recent variants). Vero E6 cells infected with each strain showed clear specific fluorescence with mAb 1G4, with no significant difference between genotype I and genotype II strains. No fluorescence was detected in the negative control (Figure 4). These results indicate mAb 1G4 targets a highly conserved epitope on gB, unaffected by PRV strain variation. Thus, it has broad cross-reactivity among genotype I, classic genotype II, and variant genotype II strains, with important application value in PRV detection and conserved epitope research.

### 3.5 Application of FITC-labeled mAb 1G4 in tracking PRV infection

**FIGURE 5:**
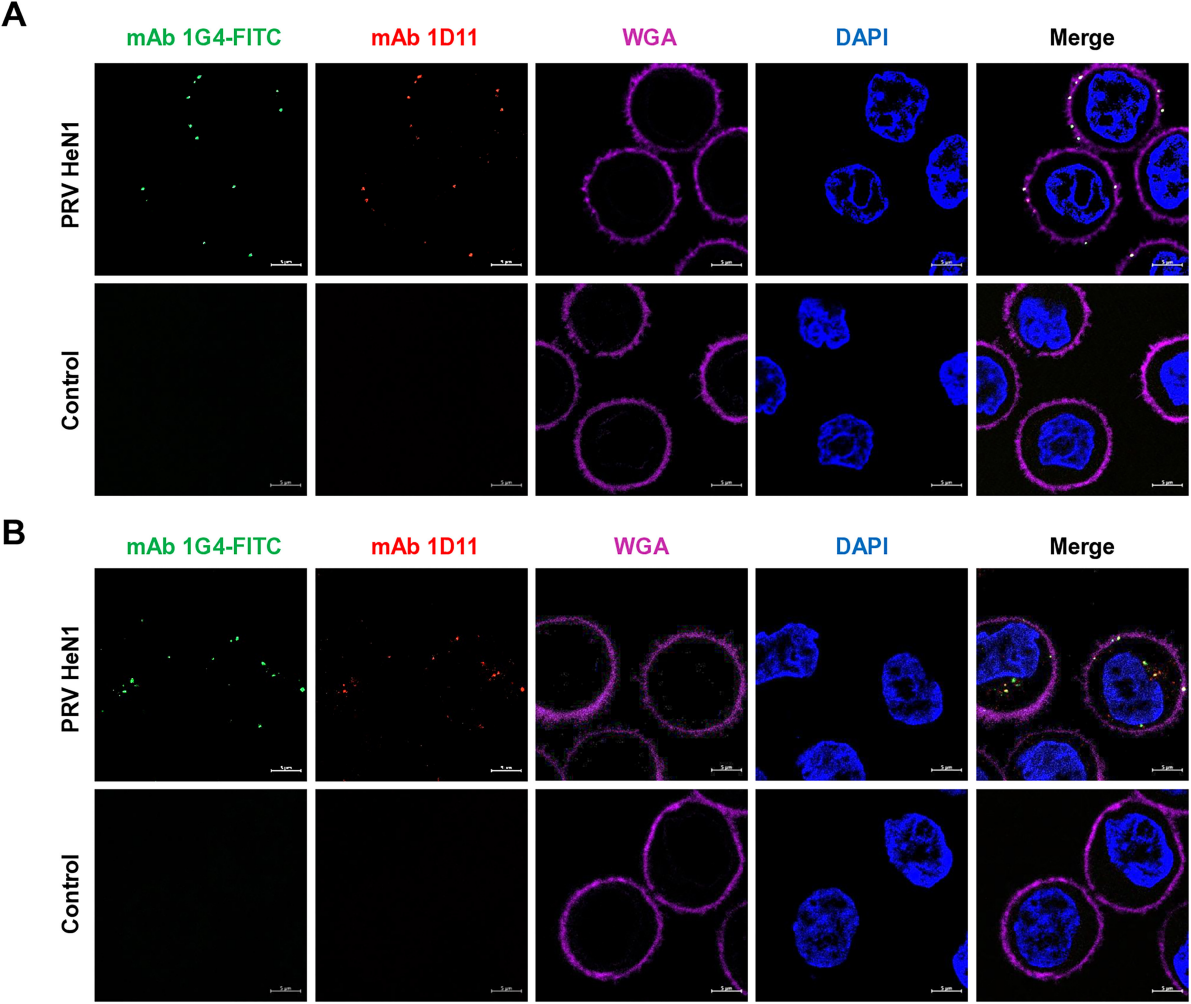
Application of FITC-labeled mAb 1G4 in tracking PRV adsorption and internalization. **(A)** Visualization of PRV adsorption on HeLa cells at 4 °C. mAb 1G4-FITC (green) labels viral gB; mAb 1D11 and Alexa Fluor 568 (red) label viral gD; WGA (violet) stains cell membrane; DAPI (blue) stains cell nuclei; Merge: co-localization of two viral glycoproteins. Scale bar = 5 μm. **(B)** Visualization of PRV internalization into HeLa cells at 37 °C. mAb 1G4-FITC and mAb 1D11 were used to trace intracellular virions after virus entry. Scale bar = 5 μm.

To expand the application scope of mAb 1G4, we labeled the purified mAb 1G4 with fluorescein isothiocyanate (FITC) to prepare FITC-labeled mAb 1G4 (mAb 1G4-FITC). This labeled antibody can be directly used for fluorescent tracking of PRV infection without the need for secondary antibodies, which simplifies the experimental operation and improves detection efficiency. We applied mAb 1G4-FITC to track the early stages of PRV infection, including the viral adsorption and internalization stages, using HeLa cells (a human cervical carcinoma cell line highly susceptible to PRV) as host cells. In the PRV adsorption assay, HeLa cells were incubated with PRV HeN1 strain under conditions that allow viral adsorption but inhibit internalization, ensuring viral particles only attach to the cell surface. After removing unadsorbed viral particles, the cells were incubated with mAb 1G4-FITC. The gD-specific monoclonal antibody 1D11 (red) was used as a co-localization control, as gD is another important PRV envelope protein involved in viral adsorption. Confocal images showed that mAb 1G4-FITC clearly labeled viral particles on the surface of HeLa cells, with obvious co-localization of green and red fluorescence, confirming that the labeled mAb 1G4 can specifically recognize the gB protein on PRV particles and accurately track the viral adsorption process (Figure 5A).

In the viral internalization assay, HeLa cells were first incubated with PRV HeN1 strain to allow viral adsorption, then unadsorbed virus was washed away, and the cells were cultured under conditions that promote viral internalization into host cells. After incubation, the cells were fixed, permeabilized, blocked, and incubated with mAb 1G4-FITC. The results showed that mAb 1G4-FITC detected obvious green fluorescence signals inside HeLa cells, indicating that viral particles had been internalized into the cells (Figure 5B). These results demonstrated that mAb 1G4-FITC has good specificity and fluorescence activity, and can be used to dynamically trace the early stages of PRV infection, providing a powerful tool for researching the molecular mechanism of PRV entry into host cells.

## 4 Discussion

Pseudorabies virus gB is a core envelope glycoprotein that mediates viral entry, membrane fusion and cell-to-cell transmission, and acts as a dominant target for host protective immunity (Li et al., 2017; Chen et al., 2022). Given its high sequence conservation and indispensable biological functions, gB is regarded as an ideal candidate for the development of diagnostic reagents and research tools. The differential diagnosis for the future application of subunit vaccines will facilitate the elimination of the disease (Tang et al., 2024, 2025). In the present study, a stable mammalian expression system based on CHO cells was utilized to produce recombinant PRV gB protein. This eukaryotic expression strategy retains native post-translational modifications and conformational epitopes, which are frequently absent in prokaryotic expression systems, thereby effectively enhancing antigen immunogenicity and guaranteeing the quality of the prepared monoclonal antibodies.

SDS-PAGE analysis showed distinct band of purified recombinant gB. Western blot assays further verified that the target protein could be specifically recognized by anti-His tag antibody and gB-immunized mouse serum, validating its high purity and favorable antigenicity. Robust high-titer antibody responses were induced in immunized BALB/c mice, laying a solid foundation for subsequent hybridoma construction.

After cell fusion and multiple rounds of limiting dilution subcloning, four stable hybridoma cell lines secreting gB-specific mAbs were successfully screened. Among these candidates, mAb 1G4 exhibited the strongest and most stable antigen-binding activity and was therefore selected for further functional identification. Indirect immunofluorescence assay (IFA) results confirmed that mAb 1G4 specifically bound to recombinant gB in transfected HEK293T cells and endogenous native gB in PRV-infected Vero E6 cells, with no non-specific binding to uninfected cells or negative control samples. Its high specificity effectively reduces background interference and ensures high reliability in immunodetection experiments. In the future, the heavy chain type of this antibody could also be investigated, as this region is associated with antibody stability and regulates the half-life of the antibody through the neonatal Fc receptor (FcRn) (Na et al., 2025b).

Notably, mAb 1G4 possessed broad cross-reactivity against multiple PRV strains, including prevalent epidemic variants (HeN1, TJ and SC strains) and the classical Bartha K61 vaccine strain. These findings suggest that the 1G4-targeted epitope is located within a highly conserved domain of gB and rarely affected by viral antigenic variation during PRV evolution. Broadly reactive pan-PRV mAbs hold great application potential for universal detection of field isolates, intraspecies antigenic analysis among different strains, and the establishment of generalized PRV diagnostic methods.

More importantly, we successfully labeled mAb 1G4 with FITC and established a dual-color confocal imaging system combined with the previously prepared gD-specific mAb 1D11. This system achieves visualization of PRV particles at the early infection stage, covering viral surface adsorption at 4 °C and subsequent internalization into host cells at 37 °C. Simultaneous labeling of gB and gD enables the observation of spatial distribution and colocalization of two key envelope glycoproteins during viral entry, providing direct morphological evidence to elucidate the molecular mechanism of PRV invasion.

Compared with conventional biochemical detection methods, FITC-conjugated mAb 1G4 supports high-resolution, dynamic and intuitive tracking of PRV infection at the subcellular level. This novel antibody tool facilitates future investigations into PRV entry pathways, membrane fusion regulation, virus-host interaction networks and high-throughput screening of anti-PRV drugs.

In conclusion, this study generated and systematically characterized a high-specificity, broad-spectrum and stable PRV gB monoclonal antibody. The FITC-labeled 1G4 enables efficient visual tracking of PRV adsorption and internalization. Collectively, our work provides a robust and versatile antibody reagent that offers valuable support for in-depth research on PRV pathogenesis, as well as for viral infection monitoring, diagnostic kit development, and targeted antiviral research.

## Data Availability

The raw data supporting the conclusions of this article will be made available by the authors, without undue reservation.
